# *VvSWEET10* Mediates Sugar Accumulation in Grapes

**DOI:** 10.3390/genes10040255

**Published:** 2019-03-28

**Authors:** Zhan Zhang, Luming Zou, Chong Ren, Fengrui Ren, Yi Wang, Peige Fan, Shaohua Li, Zhenchang Liang

**Affiliations:** 1 Beijing Key Laboratory of Grape Science and Enology, and CAS Key Laboratory of Plant Resources, Institute of Botany, the Innovative Academy of Seed Design, the Chinese Academy of Science, Beijing 100093, China; zhangzhan712@163.com (Z.Z.); zoulm@ibcas.ac.cn (L.Z.); chongr@ibcas.ac.cn (C.R.); rfrren@126.com (F.R.); wangyi19881107@163.com (Y.W.); fanpg@ibcas.ac.cn (P.F.); shhli@ibcas.ac.cn (S.L.); 2 University of Chinese Academy of Sciences, Beijing 100049, China; 3 Sino-Africa Joint Research Center, Chinese Academy of Sciences, Wuhan 430074, China

**Keywords:** SWEET, grape, sugar accumulation, véraison, invertase, overexpression

## Abstract

Sugar accumulation is a critical event during grape berry ripening that determines the grape market values. Berry cells are highly dependent on sugar transporters to mediate cross-membrane transport. However, the role of sugar transporters in improving sugar accumulation in berries is not well established in grapes. Herein we report that a Sugars Will Eventually be Exported Transporter (SWEET), that is, *VvSWEET10*, was strongly expressed at the onset of ripening (véraison) and can improve grape sugar content. *VvSWEET10* encodes a plasma membrane-localized transporter, and the heterologous expression of *VvSWEET10* indicates that *VvSWEET10* is a hexose-affinity transporter and has a broad spectrum of sugar transport functions. *VvSWEET10* overexpression in grapevine calli and tomatoes increased the glucose, fructose, and total sugar levels significantly. The RNA sequencing results of grapevine transgenic calli showed that many sugar transporter genes and invertase genes were upregulated and suggest that *VvSWEET10* may mediate sugar accumulation. These findings elucidated the role of *VvSWEET10* in sugar accumulation and will be beneficial for the improvement of grape berry quality in the future.

## 1. Introduction

Sugar transport and partitioning are important for normal plant growth. Photoassimilate allocation in plants depends on sugar transporters to control assimilation and sugar transport to various tissues and cells effectively [1]. Most fruit crops, such as grapes (*Vitis vinifera* L.), peaches, apples, and tomatoes store soluble sugar in their fruits, and the content and composition of these sugars is a major determinant of fruit quality [2,3,4]. Hence, understanding how fruit crops control sugar translocation/storage for fruit development and how this contributes to their quality is vital. 

In general, sucrose (Suc) is the predominant carbohydrate transported from leaves to various plant tissues for long-distance translocation by Suc transporters and Sugars Will Eventually be Exported Transporter (SWEET) efflux proteins [5,6]. Then, Suc or hexoses are taken up into sink cells by disaccharide transporters or monosaccharide transporters, especially those of the root, young leaves, seeds, and fruits [6,7]. Hence, these sink cells are equipped with at least one or more sugar transporters. The uptake of sugars into cells has been intensely studied in terms of the molecular and physiological basis, and many key sugar transporters have been identified in plants [6]. In Arabidopsis, 9 Suc transporters and 53 monosaccharide transporters have been identified; these transporters belong to the major facilitator superfamily (MFS) that acts as a H^+^-dependent sugar antiporter [8,9,10]. Numerous sugar transporter genes have also been reported in fruit crops [11,12,13,14]. Typically, Suc is imported into the cytosol by Suc transporters, such as Arabidopsis *AtSUC1*/*AtSUC2*, which is an apoplasmic loader [5,15], barley *HvSUT1* and *HvSUT2*, which affect Suc homeostasis during grain filling [16], pear *PbSUT2*, which affects the Suc content in sink cells [17], and grape *VvSUC11* and *VvSUC12*, which affect sugar accumulation in the berry [11]. Monosaccharide transporters regulate the cytosolic and vacuolar concentrations of reducing sugars [10]. After the hydrolysis of Suc by invertases, glucose (Glc) and fructose (Fru) are imported into target cells [18]. The sugar transport protein (STP) and the tonoplast monosaccharide transporter (TMT) represent the best characterized family of hexose transporters (HTs), that is, *AtSTP1* plays a major role in the uptake of extracellular sugars [19], tomato STP (*LeHT1*–*3*) genes by RNAi-mediated knockdown decrease fruit hexose accumulation [20], and *VvHT1* is involved in the retrieval of monosaccharides at the early stages of grape development [21]. *AtTMT1*/*AtTMT2* alter cellular sugar partitioning to promote sugar accumulation in Arabidopsis [22,23].

In addition to numerous studies on the mechanism of the major facilitator superfamily in sugar translocation/storage, SWEETs, which comprise a novel family of sugar transporters, were identified in plants, animals, and bacteria [5,24]. SWEETs are conserved throughout the plant kingdom, and the plant genomes contain a large number of SWEET paralogs, which are differentially expressed and serve various functions [25,26,27]. The roles of SWEETs in plants include phloem loading, nectar secretion, seed filling, and acting as the target of extracellular pathogens [5,28,29,30,31,32,33]. Many SWEET members have been functionally characterized as candidate genes for maintaining sugar homeostasis in sink tissues. The corn and rice *SWEET4* gene is a key player for hexose transport during seed filling and contributes to sink strength [31]. The knockout of *OsSWEET11* leads to defective grain filling in rice [34]. Arabidopsis *SWEET11*, *12*, and *15* exhibit specific expression patterns in developing seeds and mediate the step of Suc efflux from the seed coat to the embryo. Arabidopsis also showed severe seed defects in *sweet11/12/15* triple mutants [32]. Two vacuolar sugar transporters, namely, *AtSWEET16* and *AtSWEET17*, mediate Fru transport in roots and leaves [7]. In cultivated fruit crops, the tomato *SlFgr* gene encodes a SWEET that determines the hexose composition of a ripening tomato fruit [35]. The grape SWEET genes were also identified and characterized; some members (i.e., *VvSWEET4*, *VvSWEET7*, *VvSWEET10*, *VvSWEET11*, *VvSWEET15*, and *VvSWEET17d*) are highly expressed in berries, thereby suggesting that *VvSWEET* performs important functions in grape berries [36].

Sugar transport and storage are highly important for the improvement in grape berry quality. Grape berries exhibit a dynamic developmental process since the onset of ripening (véraison) as a massive amount of soluble sugars accumulate in berries [36,37]. Véraison marks the start of the grape berry ripening processes; sugar accumulation, especially rapid hexose accumulation, begins during this phase [38,39,40]. In contrast to numerous studies on sugar transporters in other plants, the determining mechanism of the accumulation of high Glc and Fru concentration in grape berries remains largely unknown.

In the present study, *VvSWEET10* was highly expressed in berry and plasma membrane localization in vivo. Heterologous expression, genetic transformation, and RNA sequencing (RNA-Seq) were performed to study the molecular function of *VvSWEET10* during the sugar accumulation in grapes. Our results showed that *VvSWEET10* contributed to Glc and Fru contents and influenced the soluble sugar composition in grapes. This information can help understand the mechanism underlying sugar accumulation in fruit crops and potentially improve the grape berry quality in the future.

## 2. Materials and Methods

### 2.1. Plant Materials

*V*. *amurensis* and *V*. *vinifera* L ‘Chardonnay’ and ‘Cabernet Sauvignon’ respectively were used in this study. *V. amurensis* was used for transgenic grapevine calli. Micropropagated *V*. *amurensis* was grown on half-strength Murashige and Skoog (MS; 1/2 MS supplemented with 30 g/L of Suc, 7 g/L of agar, and 0.2 mg/L of IBA) with light:dark (16 h:8 h) conditions at 26 °C. Chardonnay was used for gene expression analysis, and its seedlings were grown in the grape germplasm resource garden of the Institute of Botany, the Chinese Academy of Sciences, Beijing. The different organs and tissues (i.e., roots, stems, leaves, flowers, tendrils, and berries at fruit setting), before veraison, veraison, post veraison, and at the ripening stages of Chardonnay were sampled for further analysis. Meanwhile, Cabernet Sauvignon berry suspension cells were used for sugar treatments, and the culture conditions were carried out as described by Zhang et al. [41]. For sugar starvation treatments, the berry suspension cells were cultured in liquid B5 medium (B5 basal medium supplemented with 30 g/L of Suc, 2.5 g/L of hydrolyzed casein, 0.2 mg/L of KT, 0.1 mg/L of NAA). After 5 days of growth (logarithmic growth phase of cells), the medium was replaced with sugar-free liquid B5 media for a sugar starvation condition. Subsequently, the expression patterns of *VvSWEET10* were analyzed at 0, 6, 12, and 24 h.

### 2.2. RNA Extraction and Quantitative Real-Time PCR (qRT-PCR) Analyses

RNA extraction and qRT-PCR were performed as described by Zhang et al. [41]. The clustering of *VvSWEET* gene expression was constructed by HemI (http://hemi.biocuckoo.org/). Total RNA was extracted from the different organs and tissues of Chardonnay, grapevine calli, Cabernet Sauvignon berry suspension cells, and tomatoes. qRT-PCR analyses were performed with at least three technical and two biological replicates. The primers are listed in Table S1. 

### 2.3. Cloning and Sequence Analysis

The coding sequence of *VvSWEET10* was amplified from the Chardonnay berry cDNA with gene-specific primers (Table S1). The primers were designed according to the grapevine genome sequence data (12× coverage) [42]. The homologous sequence of SWEET10 from grapes, Arabidopsis, and tomatoes was individually aligned by ESPript 3.0 (http://espript.ibcp.fr/ESPript/cgi-bin/ESPript.cgi). A phylogenetic tree analysis was constructed by MEGA5 using the parameters: Poisson model, uniform rates, 1000 bootstraps, and pairwise deletion. The SWEET protein sequences from grapes, Arabidopsis, and tomatoes were as described by previous sequence databases [24,35,36].

### 2.4. VvSWEET10 Expression Localization by β-glucuronidase and Green Fluorescent Protein (GFP) Reporter Genes

The *VvSWEET10* promoter fragments in a 1945 bp upstream region of *VvSWEET10* were amplified from Chardonnay genomic DNA using the primer sets (*VvSWEET10*pro-F and *VvSWEET10*pro-R, Table S1) and Phusion polymerase (KOD, TOYOBO Co., Ltd, Osaka, Japan). The PCR fragment was digested with *Hind III* and *Xba I* and then cloned in front of the *GUS* gene to replace the 35S promoter in the binary vector pBI121. Tomato seeds (*Solanum lycopersicum* cv. Micro-Tom) were purchased from Ball Horticultural Company (https://www.ballhort.com/) and used as transgenic donors. The *pBI121-VvSWEET10pro-GUS* construct was transformed into the *Agrobacterium tumefaciens* strain EHA105. The tomato was transformed according to a previous report [43]. Tomato plants were grown at 26 °C with a light:dark (16 h:8 h) condition. Fruit ripening stages were determined by the day after flowering (DAF). The expression of the *GUS* reporter gene was analyzed via histochemical staining. Then, the samples of transgenic tomatoes and the wild type (WT) were incubated with GUS staining solution for 4–6 h at 37 °C. After staining, the samples were cleared by 70% and 95% ethanol.

To analyze the subcellular localization of *VvSWEET10*, we amplified the whole coding sequence of *VvSWEET10* using gene specific primers (*VvSWEET10*G-F and *VvSWEET10*G-R) and subcloned into the *EcoRI* and *KpnI* sites of pEZS-NL vector to generate *VvSWEET10–GFP* fusion proteins. Then, the *35S:VvSWEET10-GFP* and the control (CK) *35S:GFP* constructs were transiently expressed in protoplasts from a tobacco (*Nicotiana benthamiana*) leaf. Transfection was performed as described by Yoo et al. [44] and Zhang et al. [41]. Fluorescence images were visualized as described previously [41].

### 2.5. Complementation of the Yeast Mutant EBY.VW4000

Functional complementation experiment was performed using hexose transporter-deficient *Saccharomyces cerevisiae* EBY.VW4000 as described previously [45]. The coding sequence of *VvSWEET10* was cloned into the yeast expression vector pDR196. The *pDR196-VvSWEET10* construct or the empty vector pDR196 was introduced into EBY.VW4000 according to the previously reported method [46]. Transformants were selected on a synthetic deficient medium (2% maltose) without uracil (SD [-Ura] medium). For complementation growth assays, selected transformant cells were grown in liquid SD-Ura medium to an optical density at 600 nm of 0.6. Then, serial dilutions (OD600 = 1, 0.1, 0.01, 0.001) were plated in solid SD (-Ura) medium supplemented with 2% maltose (as the CK) or 2% Glc, 2% Fru, and 2% Suc as sole C sources. Then, the cells were grown for 3 days at 30 °C, and images were obtained. The primers are listed in Table S1.

### 2.6. VvSWEET10 Overexpression in Grapevine Calli and Tomato

The whole coding sequence of *VvSWEET10* were cloned into the binary vector pSAK277 under the control of the 35S promoter. The resulting plasmids *pSAK277*–*VvSWEET10* and the empty vector pSAK277 were transformed into a *V*. *amurensis* petiole according to the method of Zhao et al. [47]. Then, the transformants were identified by PCR and qRT-PCR (Figure S3). Four highly expressed transgenic calli T1 lines were obtained, and three highly expressed transgenic calli lines (i.e., L1, L2, and L4) lines were used in this study.

To investigate the functions of *VvSWEET10* in fruit and transgenic tomatoes further, we generated plants according to the method of a previous report [43]. Binary plasmids *pSAK277*–*VvSWEET10* were transformed into tomatoes using the *A. tumefaciens* strain EHA105. The transgenic tomato plants were confirmed by detecting *VvSWEET10* and *NPTII* with gene-specific primers (Table S1), and two highly expressed transgenic tomato plant T0 lines were obtained for further physiological analyses (Figure S8).

### 2.7. Sugar Determination

The frozen powders of grape or tomato material (1 g fresh weight) were homogenized in 6 mL of deionized water for 2 h with intermittent mixing after centrifugation (5000 g, 15 min, 4 °C). The supernatants were decanted and passed through a SEP-C18 cartridge (SBAB-57063, SUPELCO) and filtered through a 0.22 μm Sep-Pak filter [48]. Soluble sugars were analyzed using a Waters 2695 HPLC system with a Waters 2414 refractive index detector. Measurement was performed according to the method of Chen et al. [48].

### 2.8. RNA-Seq

Samples were collected from *VvSWEET10*-overexpressing (i.e., L1, L2, and L4) and empty vector transgenic calli lines. The samples were immediately frozen in liquid nitrogen and kept at −80 °C. RNA extraction, RNA-Seq library construction, and sequencing were performed by the Shanghai Majorbio Biomedical Technology Co., Ltd. (Shanghai, China; www.majorbio.com) using an Illumina HiSeq 4000 system.

The RNA-Seq raw reads were filtered using the FASTX-Toolkit (http://hannonlab.cshl.edu/fastx_toolkit/). Then, the filtered clean reads were analyzed according to the method described by Trapnell et al. [49]. The reads were mapped onto the *V. vinifera* reference genome (http://www.ensembl.org/index.html) by using TopHat software (version 2.0.14, http://ccb.jhu.edu/software/tophat). Then, Cufflinks was used to identify differentially expressed genes (DEGs). False discovery rate (FDR) was calculated using the method of Benjamini and Hochberg [50]. The FDR-adjusted *P* < 0.01 was thought to be DEGs. Fragments per kilobase of exon model per million mapped reads (FPKMs) were used to quantify the transcript abundance. Gene Ontology (GO) enrichment was performed using the agriGO analysis tool (http://bioinfo.cau.edu.cn/agriGO/analysis.php) with ensemble GO annotations (http://plants.ensembl.org/index.html). All RNA-Seq data from this study were available from the NCBI Gene Expression Omnibus under the accession number GSE124798.

## 3. Results

### 3.1. Phylogenetic and Expression Analyses of VvSWEET Genes in Grape

**Figure 1:**
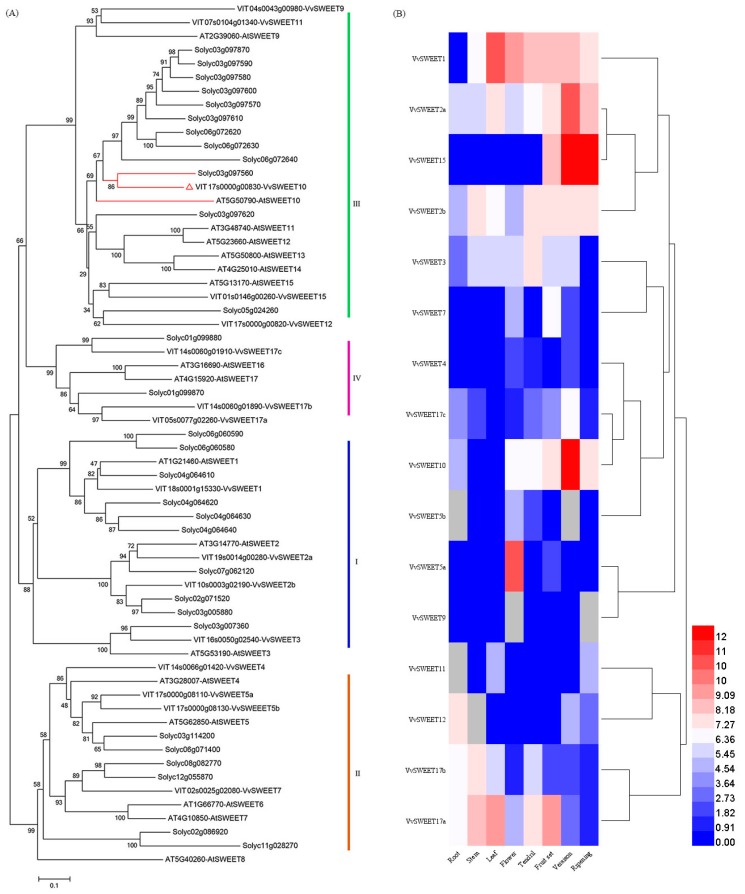
Phylogenetic and expression analyses of *VvSWEET* genes. (**A**) Amino acid sequences from grapes (*Vitis vinifera*), tomatoes (*Solanum lycopersicum*), and Arabidopsis. Evolutionary analyses were conducted in MEGA5 using the neighbor-joining method. *VvSWEET10* (red) is a member of group III. (**B**) Heat map showing the expression level of *VvSWEET* genes in grapes. qRT-PCR analyzed the relative RNA transcription of *VvSWEET* in grapes, and the gene expression level in different organs showing the highest CT values was set to 1. The expression levels presented in the heat map were log2-based. The color scale represents transcript abundance in which blue to red represents a change in the expression level from low to high.

A phylogenetic tree analysis indicated that four subclades of SWEET proteins were identified in the tree (Figure 1A), which was consistent with a previous report [36]. *VvSWEET10* belongs to clade III and is homologous to Arabidopsis *AtSWEET10* (*at5g50790*) and tomato *Solyc03g097560* (Figure 1A). The multisequence alignment analysis indicated that their amino acid sequence had a high degree of homology (Figure S1). Arabidopsis clade III SWEETs can transport disaccharides and hexoses [25]. Although *AtSWEET10* can transport Suc across the plasma membrane, it can also be induced by a pathogen; however, the diverse function of *SWEET10* in plants still needs to be characterized [5]. To explore the role of SWEETs in grapes further, we reanalyzed the *VvSWEET* expression in different Chardonnay organs (i.e., roots, stems, young leaves, tendrils, flowers, and berries). qRT-PCR results showed that numerous *VvSWEET* genes (e.g., *VvSWEET1*, *VvSWEET2a*, *VvSWEET2b*, *VvSWEET10*, *VvSWEET15*, and *VvSWEET17a*) displayed higher expression in grape berries than those in other organs (Figure 1B). The expression pattern of *VvSWEET2a*/*10*/*15* was also closely related to the different stages of berry development. These results indicate that *VvSWEET* genes may play an important role during grape berry development.

### 3.2. Tissue-Specific VvSWEET10 Expression

**Figure 2:**
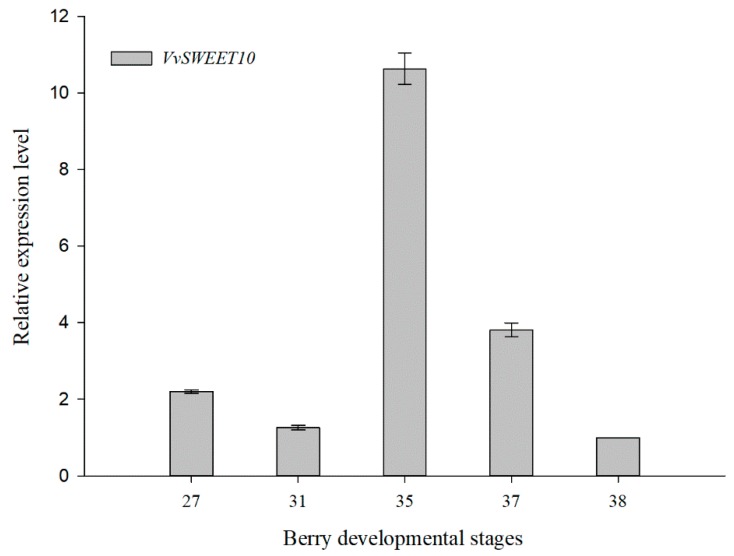
Developmental expression patterns of *VvSWEET10* in grapes. The five developmental stages (i.e., E-L stages 27, 31, 35, 37, and 38) of the berry were used for qRT-PCR. The results are presented as means ± SD (n = 3).

Grape berry development undergoes a dynamic process that contains two successive sigmoid cycles; véraison marks the beginning of the second cycle, and the rapid sugar accumulation is a major event [37]. According to the qRT-PCR information, *VvSWEET10* was strongly expressed in véraison (Figure 1B). Hence, we focused on the spatiotemporal expression pattern of *VvSWEET10* during berry development (Figure 2). The five stages of berry development from fruit set (E-L stage 27) and berry pea size (E-L stage 31) to véraison (E-L stages 35/37) and berry ripening (E-L stage 38) were selected according to the modified E-L system [51]. The time-course expression analysis of *VvSWEET10* showed that its transcripts were highly expressed at the E-L stage 35 (berries begin to gain color and enlarge) and the expression continued to decrease until the berry harvest/ripe stage (E-L stage 38).

To elucidate the expression pattern of *VvSWEET10* further, we generated transgenic tomato plants expressing a *GUS* gene, which was driven by the *VvSWEET10* promoter (*VvSWEET10pro::GUS*). The histochemical analysis of GUS expression was conducted at the breaker stage because *VvSWEET10* was highly expressed at the onset of ripening of the grapes. The GUS staining of transgenic tomato plants revealed that the *VvSWEET10* promoter became active at the breaker stage (tomato fruit showed some pink color at the onset of tomato ripening), and GUS activity was observed in the pulp, vascular bundle, and around the seed (Figure S2). After extending the staining, *GUS* was also expressed in the flowers, but no GUS activity was detected in other organs (data not shown).

### 3.3. Subcellular VvSWEET10 Localization

**Figure 3:**
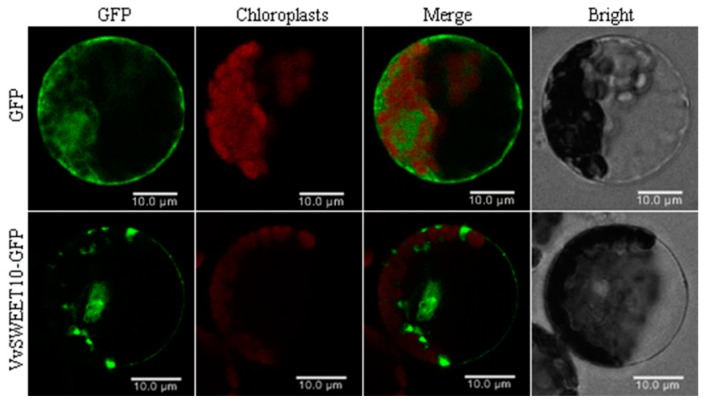
Subcellular localization of *VvSWEET10* proteins. The transient expression of *VvSWEET10*-GFP and free GFP (as a control) under the control of the 35S promoter in tobacco protoplasts. The fluorescent signal was imaged by confocal microscopy, GFP green fluorescence, and chlorophyll autofluorescence; bright-field images and merged images are shown. Scale bars = 10 μm.

To investigate the subcellular *VvSWEET10* localization, we cloned the entire coding sequence of *VvSWEET10*. The *VvSWEET10* fused with the GFP coding sequence driven by the *Caulifower mosaic virus* 35S promoter and transiently expressing the recombinant constructs in tobacco protoplasts and vector pEZS-NL containing *GFP* as a control group. Confocal images from the transient expression revealed that the *VvSWEET10*-GFP fusion protein was predominantly present at the plasma membrane of tobacco protoplasts, while GFP fluorescence from *VvSWEET10*-GFP was also observed on the reticular formation and around the nucleus. Meanwhile, the control group GFP fluorescence was distributed in the whole cell (Figure 3). These results indicate that *VvSWEET10* functioned predominantly at the plasma membrane of plant cells.

### 3.4. Functional VvSWEET10 Characterization by Heterologous Expression in Yeast EBY.VW4000

**Figure 4:**
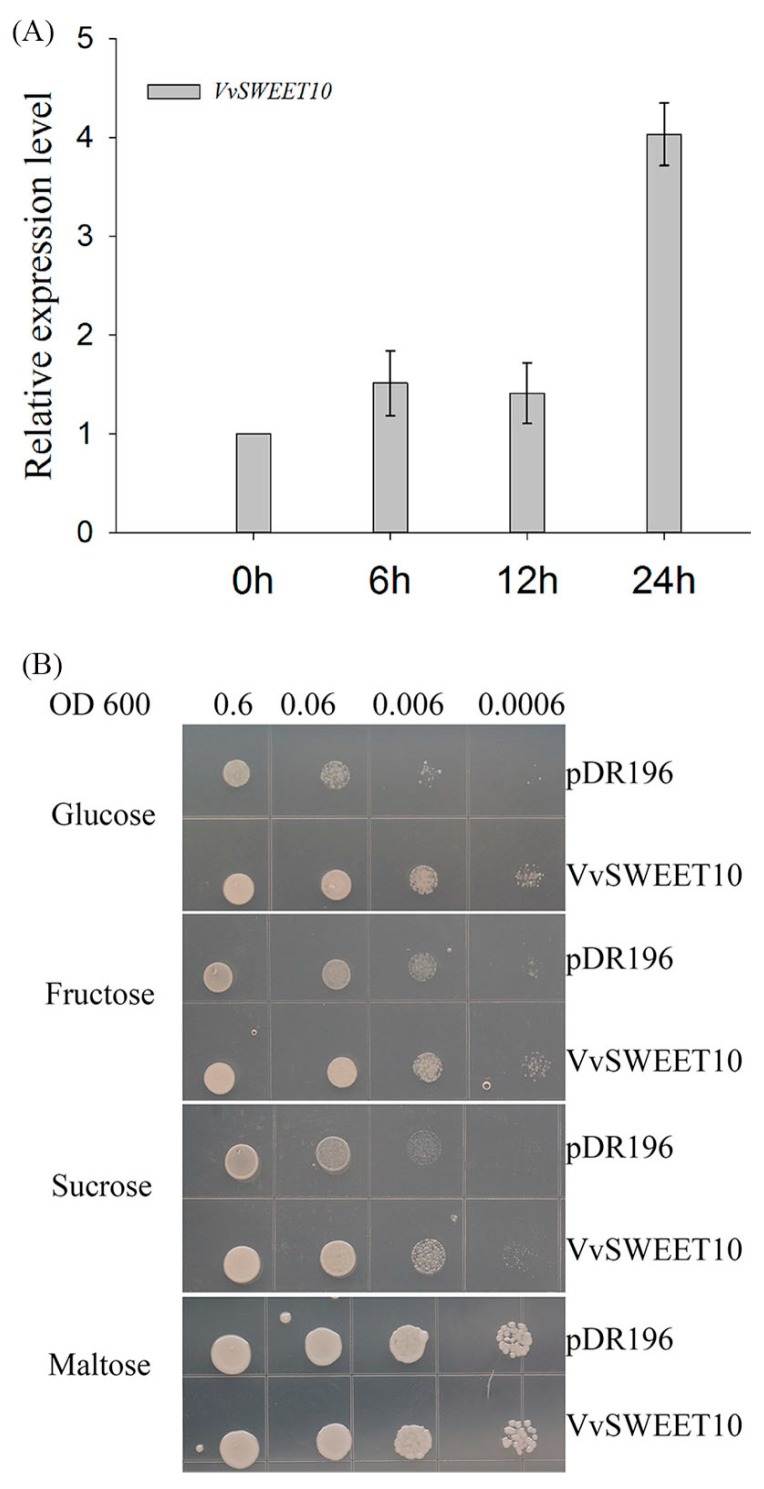
Functional characterization of *VvSWEET10*. (**A**) Low sugar supply stimulates the expression of *VvSWEET10* in grape berry suspension cells. Berry suspension cells were cultured in a sugar-free medium for a starvation condition, and the transcript levels of *VvSWEET10* were analyzed within 24 h. (**B**) Complementation of yeast EBY.VW4000 with *VvSWEET10*; drop tests were used to observe the cell growth on SD (-Ura) media supplemented with 2% maltose, 2% sucrose, 2% fructose or 2% glucose. Cells were grown at 30 °C for 3 days.

Some sugar transporters have dual functions as a sugar carrier and sensor, and changing the sugar levels affect the expression of the sugar transporter [7,22,52,53]. Assuming that *VvSWEET10* is a sugar transporter, we investigated the effect of changing sugar levels on the expression of *VvSWEET10* (Figure 4A). The results indicated that a starvation-inducible expression pattern of *VvSWEET10* was observed under the sugar starvation condition, an increase in the *VvSWEET10* transcriptional level was observed from 6 to 24 h (Figure 4A). The *VvSWEET10* expression level was significantly induced by the low intracellular and extracellular sugar levels. This result indicates that *VvSWEET10* is a functional sugar transporter.

To confirm the function of *VvSWEET10* as a sugar transporter further, we analyzed the yeast mutant EBY.VW4000 (Figure 4B). EBY.VW4000, which cannot grow on monosaccharides but can grow on a medium containing maltose, lacks 20 endogenous HT genes [45]. Following the expression of *pDR196-VvSWEET10* and the empty yeast expression vector pDR196 in EBY.VW4000, both transformant lines could grow on maltose (Figure 4B); the yeast with *VvSWEET10* was also cultured in the synthetic media supplemented with Glc, Fru, or Suc as the sole C source. The drop test clearly showed that *pDR196-VvSWEET10* expression restored the EBY.VW4000 growth on the medium containing Glc or Fru and grew slightly on the medium containing Suc (Figure 4B), thereby suggesting that *VvSWEET10* may be able to transport Glc and Fru and has an affinity for hexoses. Therefore, these results suggest that *VvSWEET10* can act as a sugar transporter in yeast EBY.VW4000.

### 3.5. Increase in Glc and Fru Accumulation in Grapevine Calli Induced by VvSWEET10 Overexpression

**Figure 5:**
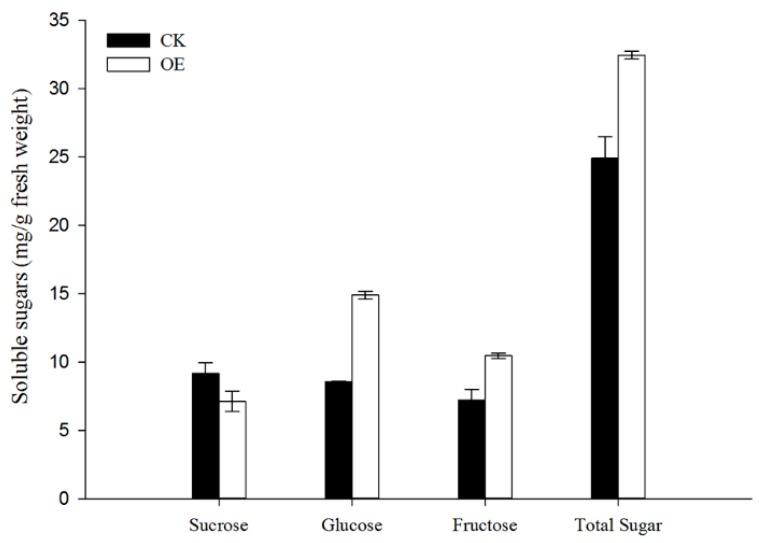
*VvSWEET10* overexpression increases sugar accumulation in grapevine calli. The sugar contents of *VvSWEET10*-overexpressed calli (OE) and the control (CK) were analyzed by HPLC. Results are shown as means ± SD (*n* = 2).

*VvSWEET10* was strongly expressed in the véraison of grapes, thereby indicating that *VvSWEET10* may affect the sugar content during berry development. To investigate if *VvSWEET10* can facilitate sugar accumulation as a sugar transporter, we generated transgenic grapevine calli from *V*. *amurensis* petiole explants and compared the *VvSWEET10*-overexpressing (OE) lines with the control (CK) lines. Four overexpression transgenic lines were confirmed by the qRT-PCR analysis (Figure S3), and the *VvSWEET10* expression level in the CK was extremely low (high CT value). This result indicated that *VvSWEET10* is a tissue-specific gene. Two representative lines of transformants (L1, L4) were selected for soluble sugar analysis, and the empty vector was used as the CK. This analysis showed significant differences in the soluble sugar levels between the *VvSWEET10*-overexpressing lines and the CKs (Figure 5). The Glc, Fru, and total sugar contents in the overexpression grapevine calli were significantly higher than those in the CK, although there were no significant changes in Suc content (Figure 5). These results indicate that overexpressing *VvSWEET10* can increase hexose accumulation in grape tissues.

### 3.6. Changes in Sugar Transport/Metabolism Gene Expression in VvSWEET10-Overexpressing Grapevine Calli

**Figure 6:**
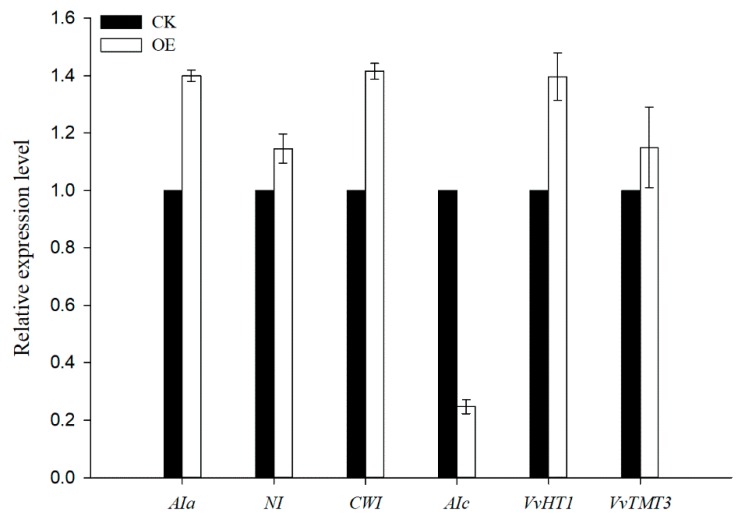
qRT-PCR validated the selection of sugar metabolism/transport DEGs. Results are means ± SD (*n* = 3).

To further understand the changes in the sugar accumulation in *VvSWEET10* overexpression, we performed RNA-Seq analysis of grapevine calli. As a result, we identified 3724 significant DEGs (22.19% of the expressed genes) in *VvSWEET10*-overexpressing grapevine calli compared with the CK (*P* < 0.01), of which 2039 genes were upregulated and 1685 genes were downregulated in the overexpressing calli, the fold change of upregulated genes ranged from 1.25 to 2198.5 and downregulated genes ranged from 1.24 to 93.97 (Supplemental Data S1). Many sugar transport/metabolism genes, including the well-characterized HT *VvHT1*, were upregulated in *VvSWEET10*-overexpressing lines (Table 1). *VvHT1* transcripts are abundant in grape berry and encode the Glc transporter [21,54]. The TMT *VvTMT3* was also significantly upregulated. The members of grape *SWEET* gene family also displayed distinct differences in our transcriptome data (Table 1, Figure S7). Five *SWEET* genes (i.e., *VvSWEET2a*, *VvSWEET4*, *VvSWEET15*, *VvSWEET17a*, and *VvSWEET17c*) were upregulated in overexpressing grapevine calli. Previous qPCR results indicated that *VvSWEET15* was strongly expressed in berries after véraison, *VvSWEET2a* and *VvSWEET17c* were highly expressed in berry véraison, and *VvSWEET17a* was highly expressed in the fruit set of berries (Figure 1B). These results indicated that the sugar transport pathway was activated after overexpressing *VvSWEET10*. Although Suc transporters exhibited an insignificant difference between the OE and CK groups, a set of plant invertase genes exhibited a significant difference between the groups (Table 1). Invertase is a key enzyme in Suc metabolism and sugar transportation [55], and functions in hydrolyzing Suc into Glc and Fru. In our RNA-Seq data, four invertase genes were significantly upregulated, and one invertase gene was downregulated in the overexpression grapevine calli (Table 1). The transcriptome data were further validated by qRT-PCR. The genes mentioned above were selected, and these genes showed consistent results between the qRT-PCR and RNA-Seq data (Figure 6). 

To obtain a global view of all significant DEGs, we next performed a GO enrichment analysis. All up- and downregulated genes were classified in one main GO, that is, cellular component, in which no genes (DEGs) were clustered in a molecular function or biological process (Figures S5, S6 and S9). As shown in Figures S5 and S6, the most significant functional groups (red maker) were related to the plasma membrane, cell wall, and intracellular part. These results indicate that increased *VvSWEET10* expression affected the cellular component modification.

### 3.7. VvSWEET10 Overexpression in Tomatoes

**Figure 7:**
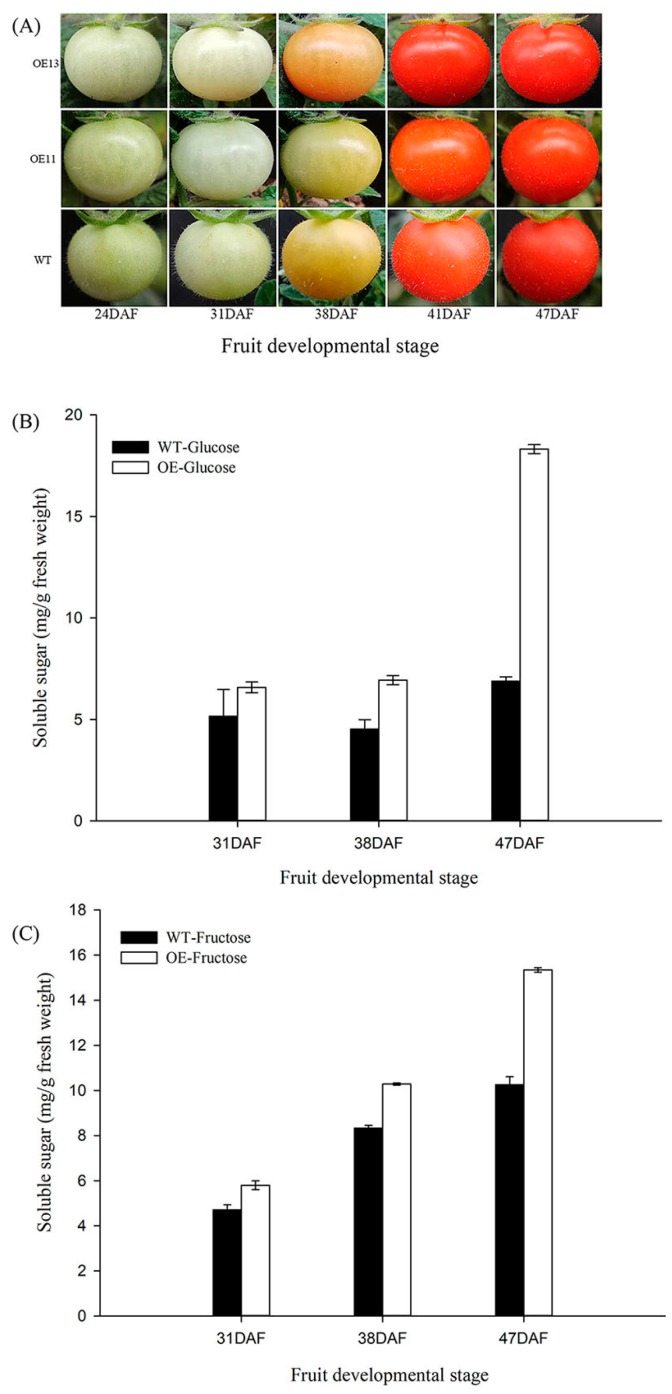
Hexose levels of transgenic lines (i.e., OE11 and OE13) and wild-type control in developing tomato fruit. (**A**) Photos of tomato fruit from *VvSWEET10*-overexpressed and wild-type (WT) lines; fruit ripening stages were determined by the day after flowering (DAF). (**B**) The glucose content in developing *VvSWEET10*-overexpressed (OE) and WT tomato fruits. (**C**) The fructose content of developing *VvSWEET10*-overexpressed (OE) and WT tomato fruits. Results are expressed as means ± SD (*n* = 3).

To improve our understanding of the function of *VvSWEET10* in berries, we generated transgenic tomato plants that overexpressed *VvSWEET10* (OE, Figure 7). Two independent OE lines were confirmed for further analysis (Figure S8). In our RNA-Seq data, some cell wall metabolism genes and sugar metabolism genes were differentially expressed (Supplemental Data S1), thereby suggesting a possible effect on fruit ripening. Hence, we compared the change in fruit color between OE and WT at 24, 31, 38, 41, and 47 DAF; the differences in the fruit-ripening process were insignificant (Figure 7A). *VvSWET10* upregulation improved the hexose level in the grapevine calli (Figure 5). Therefore, we next tested the hexose content of tomato fruit during the fruit-ripening process. Similar to those in grapes, the Glc and Fru contents in OE tomatoes were significantly higher than those in wild-type tomatoes (Figure 7B,C). These changes in sugar contents in transgenic tomato fruits suggest that *VvSWEET10* can affect the sugar composition and accumulation of other fruits.

## 4. Discussion and Conclusions

Sugar accumulation in grapes is highly dependent on the sugar transporter. The upregulation of sugar transporter genes potentially provides machinery to mediate the sugar transport pattern in berries. However, the shift mechanism of rapid sugar accumulation at the onset of ripening (véraison) remains largely unknown in grapes. In this study, we reported a grape SWEET, that is, *VvSWEET10*, which is strongly expressed in véraison, and its upregulation led to glucose improvement and fructose accumulation in grapes.

Grape clade III SWEET gene *VvSWEET10* was identified to be upregulated post-véraison [36]. Véraison marks the beginning of the ripening processes [37]. At this stage, soluble sugars continue to accumulate, the berry softens, the renewed size further increases, and rapid sugar accumulation (largely Glc and Fru) is a critical event [37,38]. The combination of qPCR analysis and histochemical analysis showed that *VvSWEET10* was preferentially expressed in véraison (Figure 1B and Figure 2), and GUS activity was observed in the pulp and vascular bundle (Figure S2). This result suggests that the *VvSWEET10* was particularly important for grapes.

Previous reports suggested clade III SWEET in Arabidopsis mainly transports Suc [5,30]. However, our experiments confirmed that *VvSWEET10* upregulation improved Glc and Fru content in grapes (Figure 5). Clade III SWEETs also play central roles in pathogen resistance; rice clade III SWEET functions as a Suc transporter and supports pathogen growth, wherein *OsSWEET11*, *OsSWEET13*, and *OsSWEET14* are targeted by pathogens [56,57,58,59]. Hence, only Suc-transporting SWEETs are the key targets of pathogens. According to Eom et al. [30], given that cytosol hexose is limited in contrast to Suc, activating a hexose SWEET may not be enough to support the growth of a pathogen population. Grape clade III SWEET expression is weakly induced by *P*. *viticola* or *E*. *necator*; and the homologous genes of *OsSWEET11*, *OsSWEET13*, and *OsSWEET14* are absent in grape clade III [36]. These results imply that grape clade III SWEET gene *VvSWEET10* is a hexose affinity transporter.

There have been numerous reports on the functional identity of SWEET in vegetative organs or sink organs [5,7,30,32,34]. Some reports extend the potential function of SWEET in sugar accumulation; the tomato *Fgr* gene encodes a plasma membrane-localized SWEET, overexpression of which leads to a modified sugar accumulation in tomato fruits [35]. Grape SWEET10 protein shares a homology with Arabidopsis *AtSWEET10* (Figure S1). *AtSWEET10* is a bidirectional sugar transporter, which is activated by an ERF transcription factor (*MaRAP2-4*) and assists in sugar accumulation in the required tissues [60]. On the basis of our result, after the expression of *VvSWEET10* in the HT-deficient yeast strain EBY.VW4000, EBY.VW4000 restored the growth on the medium containing Glc and Fru and grew slightly on medium containing Suc (Figure 4B). This finding strongly suggests that *VvSWET10* as a hexose affinity transporter possesses a broad spectrum of sugar transport functions. We also generated transgenic grapevine calli and tomato plants overexpressing the *VvSWET10* gene. *VvSWET10* overexpression produced a significant difference in the levels of soluble sugars, and the Glc, Fru, and total sugar contents were significantly increased in the transgenic grapevine calli (Figure 5). The transgenic tomato fruit also exhibited increased Glc and Fru contents during fruit ripening (Figure 7B, Figure 7C). According to these important features of *VvSWET10*, we concluded that *VvSWET10* can mediate the hexose transport, and the *VvSWET10* upregulation improved the accumulation of fruit sugar. 

Grape sarcocarp cells are strong sink cells whose vacuoles play central roles in storing soluble sugars. However, in the present study, *VvSWEET10* was predominantly located in the plasma membrane (Figure 3). Thus, the mechanism underlying the contribution of the plasma membrane SWEET to fruit sugar accumulation should be explained. A previous work revealed a shift of phloem unloading from the symplasmic to apoplasmic pathway at the onset of ripening (véraison) and the apoplasmic unloading pathway involved in the high-level accumulation of soluble sugars during berry ripening; thus, a high concentration of soluble sugar was found in the apoplasmic space of berries at the beginning of ripening [2]. The high extracellular level of soluble sugars promotes berry sugar accumulation. The level of soluble sugar in the cytosol was lower than those of the apoplasmic space and vacuole; thus, the turgor pressure gradient may be favorable for the plasma membrane SWEET to mediate the sugar transport. Our finding indicates that the *VvSEET10* expression responds to the changes in the sugar level (Figure 4A). Grape suspension cells were cultured under the sugar starvation condition. The *VvSEET10* expression was upregulated, thereby upregulating the affinity of the transporters and allowing cells to adapt to the reduction of intracellular and extracellular sugars. The GUS staining activity possessed a visible accumulation around the vascular bundle (Figure S2). Therefore, *VvSWEET10* may be important in retrieving and transporting sugar in the apoplast after the soluble sugars were unloaded from the phloem tissue at the onset of grape ripening.

Grape berries undergo stage-dependent changes during the ripening processes; véraison denotes the beginning of ripening, which is characterized by an increase in the hexose contents, softening of the berry, and a renewed increase in size [37]. An apoplasmic transmembrane pathway is involved in sugar accumulation in this stage; Suc is unloaded from the phloem, and continuous hydrolysis occurs using invertase [2]. Meanwhile, the sugar transporters capture the sugar from the apoplasmic space, and upregulating the transporters may be conducive to sugar accumulation. Our RNA-Seq analysis showed that *VvSEET10* upregulation led to the differential expression of many sugar transporter genes, including HT, TMT, ERD6-like transporter, and SWEETs, in grapevine calli (Table 1). Hence, we hypothesized that *VvSEET10*, which is strongly expressed in véraison, may directly or indirectly trigger the sugar transport process. A previous study showed that the invertase amounts and activities result in a rapid increase in véraison, which provides a possible machinery to convert Suc into Glc and Fru when soluble sugar was unloaded from the phloem to the berry apoplast [2]. Our RNA-Seq data indicated one cell wall invertase (CWI) gene, two acid invertase (AI) genes, and one neutral invertase (NI) gene were significantly upregulated in the *VvSWEET10*-overexpressed grapevine calli (Figure 6, Table 1). This finding suggests that upregulation of *VvSEET10* influenced the expression of invertase genes.

Our GO enrichment analysis also revealed that all DEGs fell into one main GO term, that is, the cellular component; most of the significantly enriched GO terms were subjected to membrane system modification (Figures S5, S6, and S9). This may be a species-specific feature in grape berries, which accumulate high levels of hexoses. The molecular mechanisms of *SWEET* genes in other organs may not be exploited by grape berries. Grapes have a renewed increase in size at véraison that will be accompanied by cell division and vacuole expansion [37]. Hence, berry osmotic strength from the contribution of soluble sugars can drive vacuole expansion and membrane modification [61]. Therefore, the GO analysis result indicates that *VvSEET10* upregulation contributes to the increase in sugar in grapes.

In conclusion, our study provided functional characterizations of *VvSEET10* during the fruit-ripening stage. *VvSEET10*, as a plasma membrane transporter, was highly expressed in véraison, which may mediate the apoplasmic transmembrane transport pathway of hexoses. Our work also highlighted the potential role of the *VvSEET10* transporter for the improvement of berry sugar accumulation.

## Figures and Tables

**Table 1:** Classification of the sugar metabolism/transport differentially expressed genes on the basis of RNA-Seq analyses.

Gene ID	Name	Log2 (Fold Change; OE/CK)	Relative Expression Level(OE/CK)	Abbreviation
VIT_02s0154g00090	Invertase	−1.60	Up-	INV(AIa)
VIT_00s2527g00010	Invertase	-Inf	Up-	INV(AIb)
VIT_16s0022g00670	Invertase	−0.50	Up-	INV(CWI)
VIT_14s0060g00860	Invertase	−0.37	Up-	INV(NI)
VIT_09s0002g02320	Invertase	0.54	Down-	INV(AIc)
VIT_11s0016g00470	Sucrose synthase	0.82	Down-	SUS
VIT_11s0118g00200	Sucrose-phosphate synthase	−0.51	Up-	SPS
VIT_18s0089g01230	Fructokinase	−0.98	Up-	FRK
VIT_15s0048g01260	Fructokinase	−1.30	Up-	FRK
VIT_01s0011g05370	Phosphoglucomutase	−0.42	Up-	PPGase
VIT_14s0108g00540	6-Phosphofructokinase	1.64	Down-	PPFTK
VIT_14s0006g02720	Hexose transporter	−0.37	Up-	HT
VIT_00s0181g00010	Hexose transporter	−0.52	Up-	VvHT1
VIT_10s0405g00050	Hexose transporter	−0.67	Up-	HT
VIT_10s0003g03930	Hexose transporter	0.75	Down-	HT
VIT_05s0020g03140	Hexose transporter	1.11	Down-	HT
VIT_07s0031g02270	Tonoplast monosaccharide transporter	−0.85	Up-	VvTMT3
VIT_10s0405g00050	Inositol transporter	−0.67	Up-	INT
VIT_10s0003g03930	Inositol transporter	0.75	Down-	INT
VIT_04s0023g01860	ERD6-like transporter	−0.40	Up-	ERD6
VIT_14s0006g02720	ERD6-like transporter	−0.37	Up-	ERD6
VIT_07s0104g00830	ERD6-like transporter	−0.61	Up-	ERD6
VIT_14s0030g00300	ERD6-like transporter	2.24	Down-	ERD6
VIT_17s0000g00830	SWEET	−11.10	Up-	*VvSWEET10*
VIT_01s0146g00260	SWEET	−2.00	Up-	VvSWEET15
VIT_14s0060g01910	SWEET	−3.21	Up-	VvSWEET17c
VIT_05s0077g02260	SWEET	−1.37	Up-	VvSWEET17a
VIT_19s0014g00280	SWEET	−0.46	Up-	VvSWEET2a
VIT_14s0066g01420	SWEET	1.21	Down-	VvSWEET4

Up- and Down- represent up- and downregulation, respectively; Inf represents OE lines specific expression; AI: acid invertase, NI: neutral invertase, CWI: cell wall invertase.

## Supplementary Materials

The following are available online at https://www.mdpi.com/2073-4425/10/4/255/s1, Figure S1: Comparison of the grape SWEET10 amino acid sequence with tomatoes and Arabidopsis, Figure S2: Tissue-specific expression of *VvSWEET1*0 in tomato fruit at the breaker stage. (A) The scheme of the vector used for tomato (*Solanum lycopersicum* cv. Micro-Tom) transformation. (B) GUS expression was detected in transgenic and wild-type tomatoes; VB, vascular bundle, P, pulp, Figure S3: *VvSWEET10* overexpression in grapevine calli. (A) Transgenic grapevine calli introduced with pSAK277-*VvSWEET10* and empty vector as a control. (B) Transgenic lines (L1–L4) were verified by q-PCR analysis. Results are expressed as means ± SD (n = 3), Figure S4: (A) Scatter plot showing the correlation of gene expression between *VvSWEET10* overexpression (OE) and control (CK). The FPKMs of genes in CK and OE were significantly highly correlated (Pearson correlation value = 0.98, *P* < 2.2 × 10^−16^). (B) Volcano plot for differential gene expression (*P* < 0.05). Dots in red represent the upregulated genes, those in green represent the downregulated genes, those in blue represent the unregulated genes, and the unexpressed genes are in yellow, Figure S5: Hierarchical tree of significant GO terms in upregulated genes, Figure S6: Hierarchical tree of significant GO terms in downregulated genes, Figure S7: Comparison with the expression level of *VvSWEET* genes in *VvSWEET10*-overexpression (OE) grapevine calli and control (CK). FPKMs were used to quantify the transcript level on the basis of the RNA-Seq data, Figure S8: NPT and *VvSWEET10* gene detection at the DNA and mRNA levels in the transgenic tomato plants. (A) *VvSWEET10*-overexpressed (OE) tomato were verified for DNA insertion. (B) *VvSWEET10* expression in tomato transgenic lines was analyzed by reverse-transcription PCR (RT-PCR) analysis in leaves. M, P, and WT represent the Maker, *pSAK277*-*VvSWEET10* plasmid, and the wild type, respectively, Figure S9: Significant GO terms involved in up- and downregulated genes (Pfdr < 0.01). All GO terms are grouped into one ontology, that is, cellular component, Table S1: Primers used in this study, Supplemental Data S1: Genes differentially expressed in *VvSWEET10*-overexpression (OE) and control group (CK) grapevine calli.
