# Supplementary material for: VvSWEET10 Mediates Sugar Accumulation in Grapes
Source: Genes (Basel). 2019 Mar 28;10(4):255. doi: 10.3390/genes10040255 (PMC6523336; doi:10.3390/genes10040255)
Supplement: Supplementary file 1 [file genes-10-00255-s001.zip › Table S1.docx]

Table S1 Primers used in this study

| Primers for real-time PCR | | | |
| --- | --- | --- | --- |
| Gene ID | Gene | Forward primer | Reverse primer |
| VIT_18s0001g15330 | *VvSWEET1* | GCCCTTCTTCTTATCACTGTTCG | CCAGATTCTTGGACTTGCCTTT |
| VIT_19s0014g00280 | *VvSWEET2a* | AGGATGTTTGGGCTGCTGAT | GAGCAGCACAACTCAAGAACC |
| VIT_10s0003g02190 | *VvSWEET2b* | TTCGTGTCGCCCATACCTAC | CCAGGTGATACAAGGGGCAT |
| VIT_16s0050g02540 | *VvSWEET3* | AGTGTTCTGCATCACCGCTA | ACTACCACCAGGGGAGAACC |
| VIT_14s0066g01420 | *VvSWEET4* | GGCTCGGACTGTGATTGGTA | ACATGCAGTTCATCACTGTGG |
| VIT_17s0000g08110 | *VvSWEET5a* | TGCCATTCACCTTGTCGTTG | GCTTCTTATCCTCCTCGTCCTTT |
| VIT_17s0000g08130 | *VvSWEET5b* | GTCATCAGGACGAGGAGCG | TTGTAGAAGGCAGCGTAGAGG |
| VIT_02s0025g02080 | *VvSWEET7* | ACCGCAGTTGGCATCCTA | GCAAGGTAGGGAACTGGTGA |
| VIT_04s0043g00980 | *VvSWEET9* | ACCATCCAAGCCACCTACCT | ACCAAGCCCAGTGATGCTAC |
| VIT_17s0000g00830 | *VvSWEET10* | CATCCTGGGCTTCGTCTTC | ATTGCTGTGCTGCTGGTTAGT |
| VIT_07s0104g01340 | *VvSWEET11* | TACGCACCAAGGACAGCC | CGCAGACCCAACCGACTA |
| VIT_17s0000g00820 | *VvSWEET12* | GGGTTTCGTCTTTGGGTTG | GACCATTTCCGCCTTCATT |
| VIT_01s0146g00260 | *VvSWEET15* | GGCACATCTGACATCCATCC | TTCCCAGTTTCTTCATCATCCT |
| VIT_05s0077g02260 | *VvSWEET17a* | GGTTTTGGTGTGGTTGTTGAA | AGCTAGAAACCCCACATCCAA |
| VIT_14s0060g01890 | *VvSWEET17b* | TCTCCTGTGCCGACGTTCTC | TTGACAGTGGCTATCAGGAGG |
| VIT_14s0060g01910 | *VvSWEET17c* | CTGGCGGCTTACTTGTCCT | AAAGCCAACATCCAATACGG |
| AY680701 | *Action1* | CTTGCATCCCTCAGCACCTT | TCCTGTGGACAATGGATGGA |
| VIT_02s0154g00090 | *AIa* | TTGGCCCGATGGAAAATGGA | AGTGTCCAACCCATTATCTTCCT |
| VIT_14s0060g00860 | *NI* | TCTAGGATAGAATCTGAGGCTGA | CAAGAAAGCGAGTGCCGATG |
| VIT_16s0022g00670 | *CWI* | CGATGGAAATTGGCGGGTTG | CCCATGCAATTCTCCGTCCA |
| VIT_09s0002g02320 | *AIc* | CCTCCAAAATCAGTCTCCCTCTTC | TCGGTTGATGTGGAATGTGCC |
| VIT_00s0181g00010 | *VvHT1* | TTATTGTGGCAACGTGCATTGG | ATAGACGCAAATGAACAGCACC |
| VIT_07s0031g02270 | *VvTMT3* | TGGGATGAGGAGAGCCTTCAG | CCACCTTCTCCACCAATCCCC |
| Primers for overexpression of *VvSWEET10* | | | |
| *pSAK277-VvSWEET10* | | CGGAATTCCTGCATTCTGTCCTTGCCCAC | GGGGTACCGGAAGTGGGAAGCCTTTGTTTTTA |
| Primers for localization of *VvSWEET10* | | | |
| *VvSWEET10G* | | CGGAATTCCATTCTGTCCTTGCCCACCT | GGGGTACCTGGGAAGCCTTTGTTCAGAGC |
| *VvSWEET10pro* | | CCCAAGCTTTGCATGGCCTGAGAAACAAGA | GCTCTAGACTCTTCAGCTGGGAGGTGGG |
| *NPT* | | TCATCTCACCTTGCTCCTGC | AAGGCGATAGAAGGCGATGC |
| Primers for complementation experiment | | | |
| *pDR196-* *VvSWEET10* | | AACTGCAGTAGAAGAAAAACCCCTTCTGC | CCGCTCGAGCAGAGCAACTATGACTTCAA |
| Primers for identification of transgenic tomato | | | |
| *RT-PCR Primers* | | GCCTTGTTTCCCATTCACCATC | CAGAGCAACTATGACTTCAAGGC |
| *NPTⅡ* | | ATTACCTTATCCGCAACTTCTTTACC | AGCCCCTGATGCTCTTCGTC |

Restriction sites are underlined.
