# Supplementary figures and images for: VvSWEET10 Mediates Sugar Accumulation in Grapes
Source: Genes (Basel). 2019 Mar 28;10(4):255. doi: 10.3390/genes10040255 (PMC6523336; doi:10.3390/genes10040255)

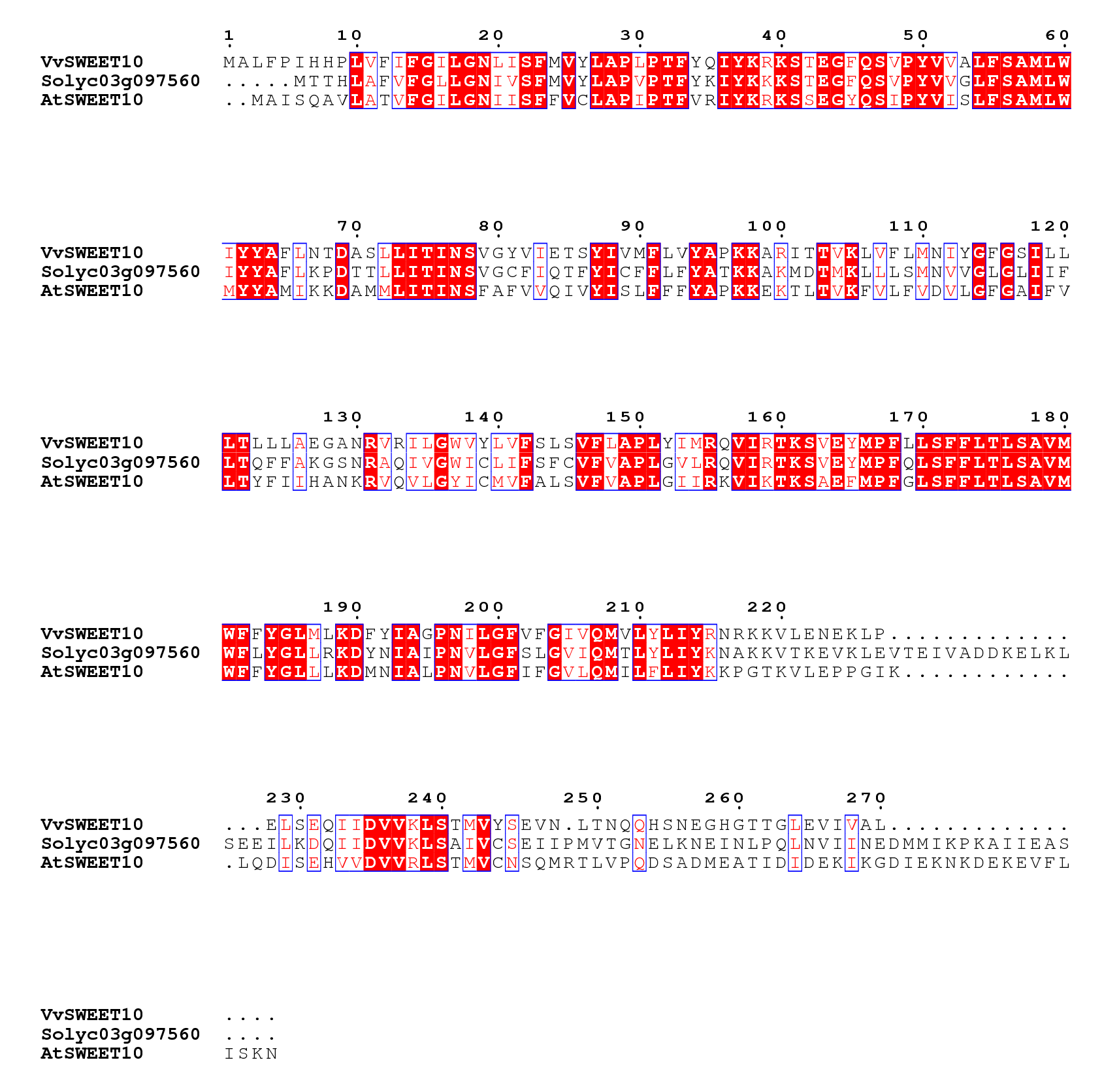

Supplement: Supplementary file 1 [file genes-10-00255-s001.zip › Fig. S1.tif]

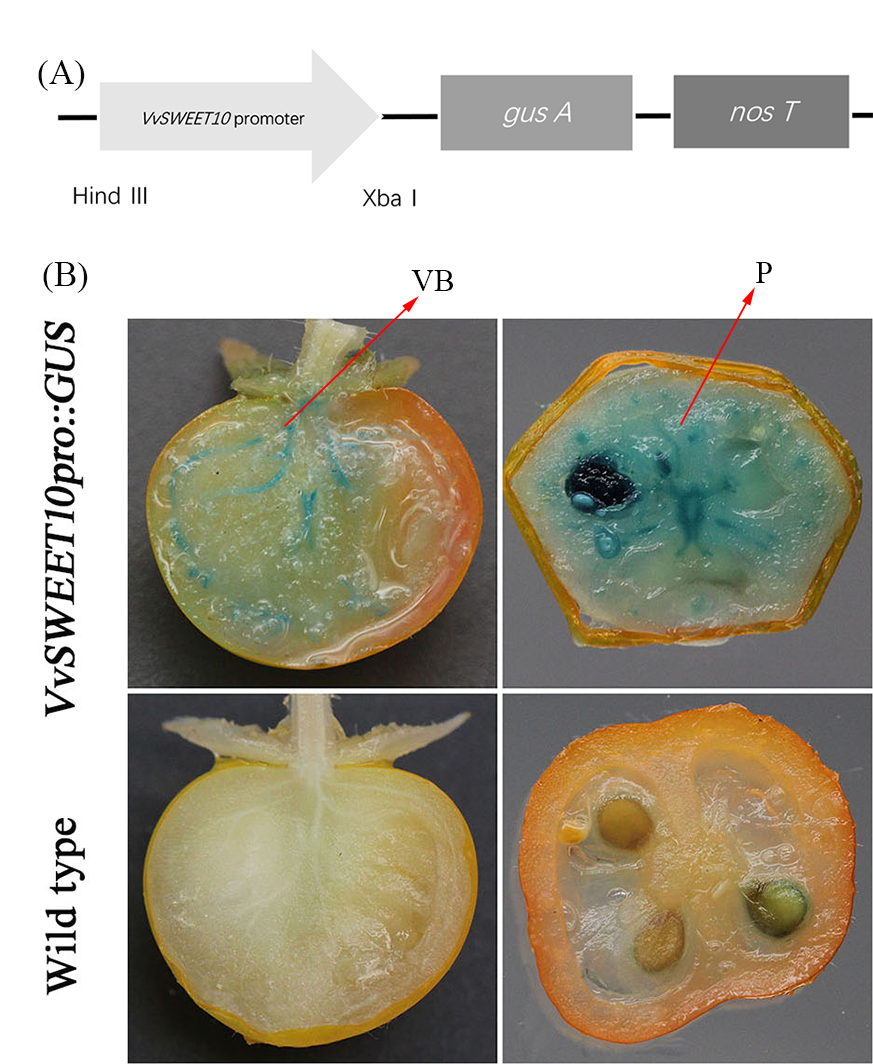

Supplement: Supplementary file 1 [file genes-10-00255-s001.zip › Fig. S2.tif]

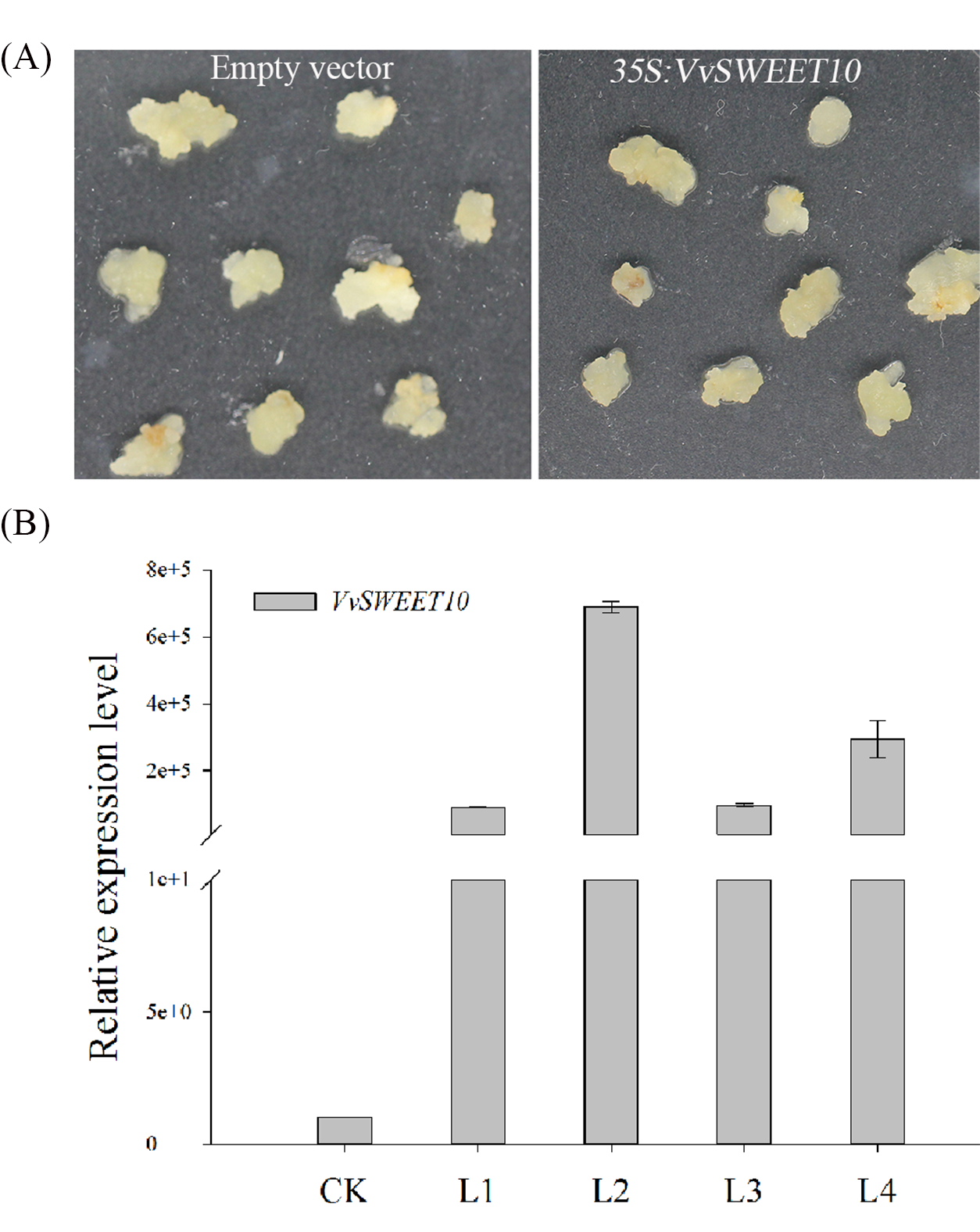

Supplement: Supplementary file 1 [file genes-10-00255-s001.zip › Fig. S3.tif]

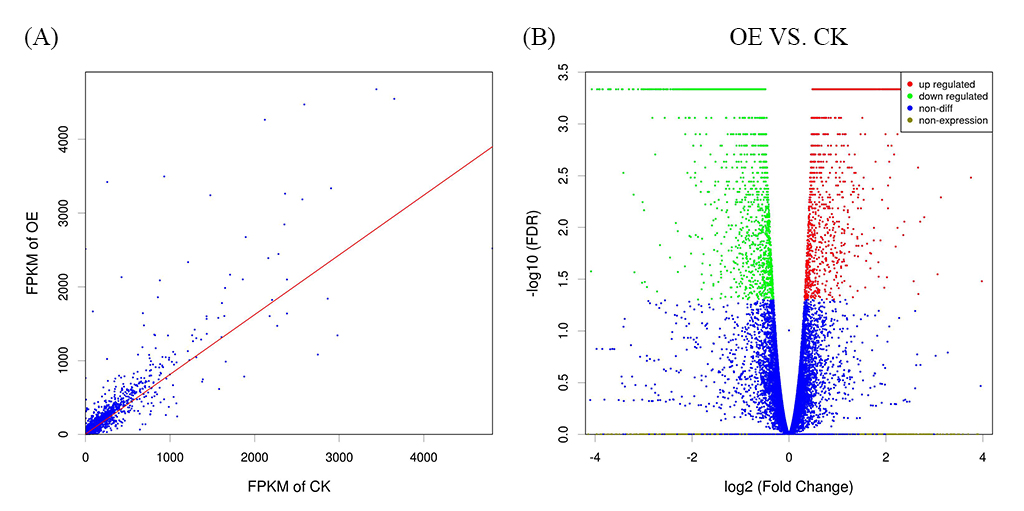

Supplement: Supplementary file 1 [file genes-10-00255-s001.zip › Fig. S4.tif]

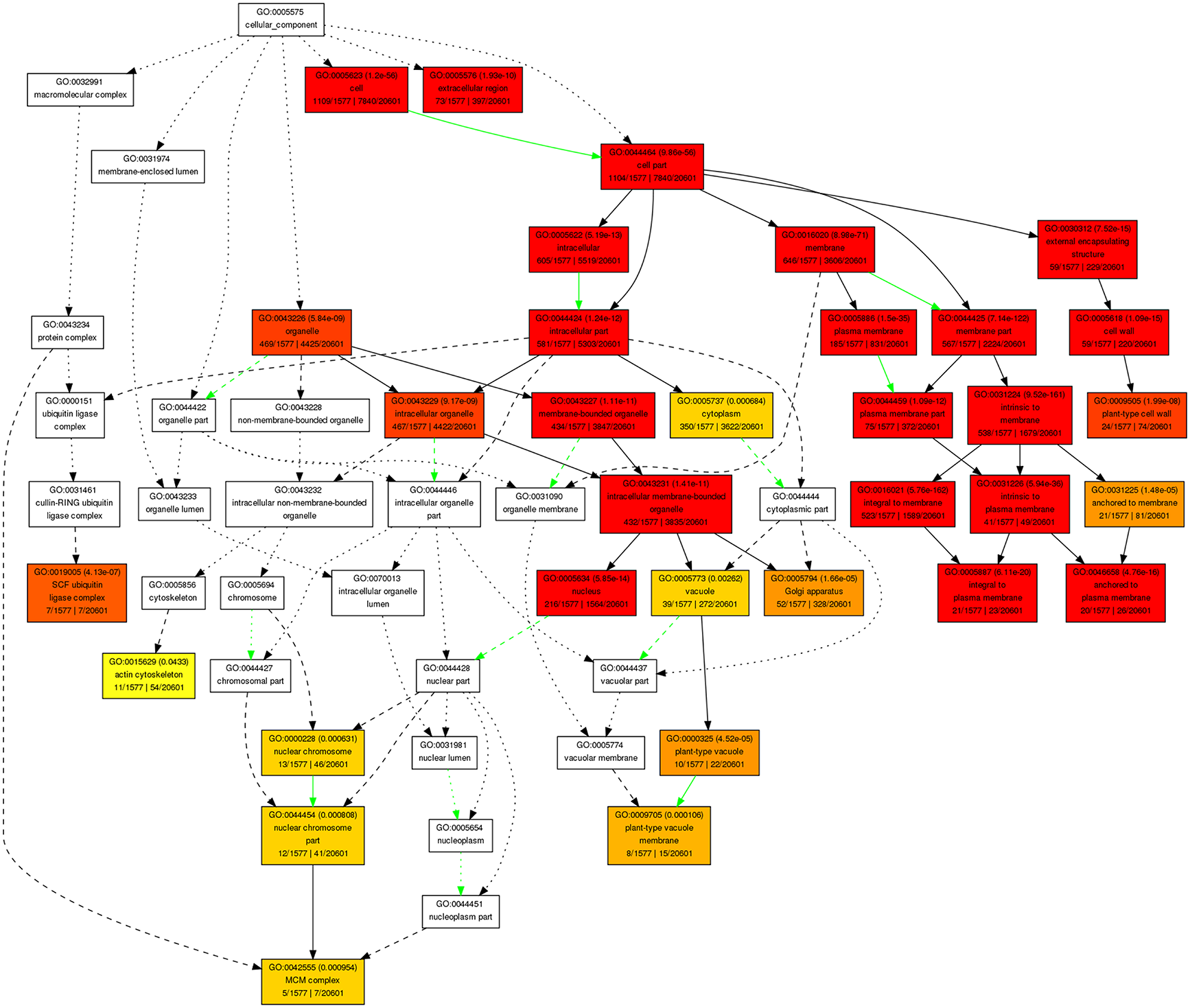

Supplement: Supplementary file 1 [file genes-10-00255-s001.zip › Fig. S5.tif]

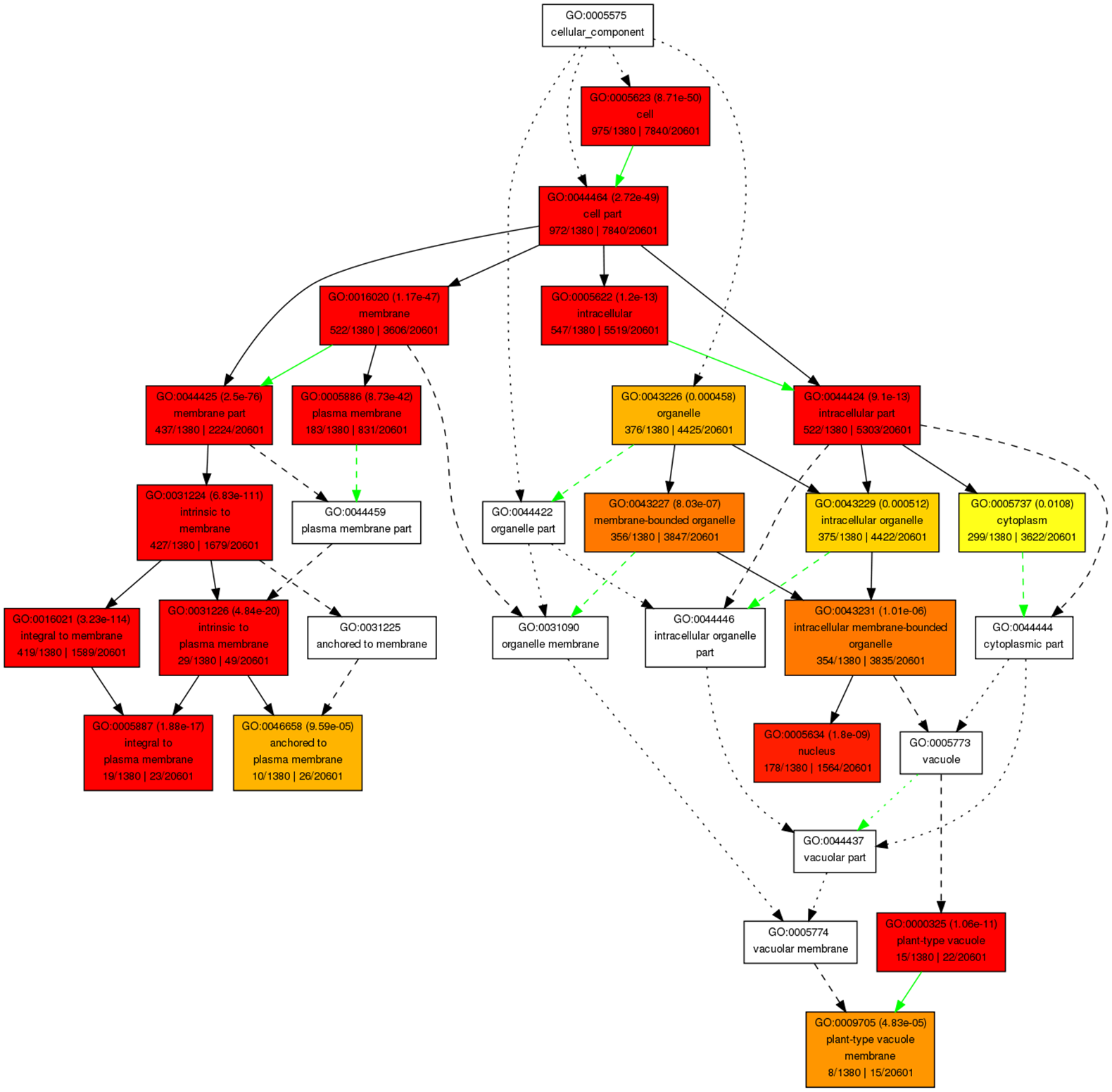

Supplement: Supplementary file 1 [file genes-10-00255-s001.zip › Fig. S6.tif]

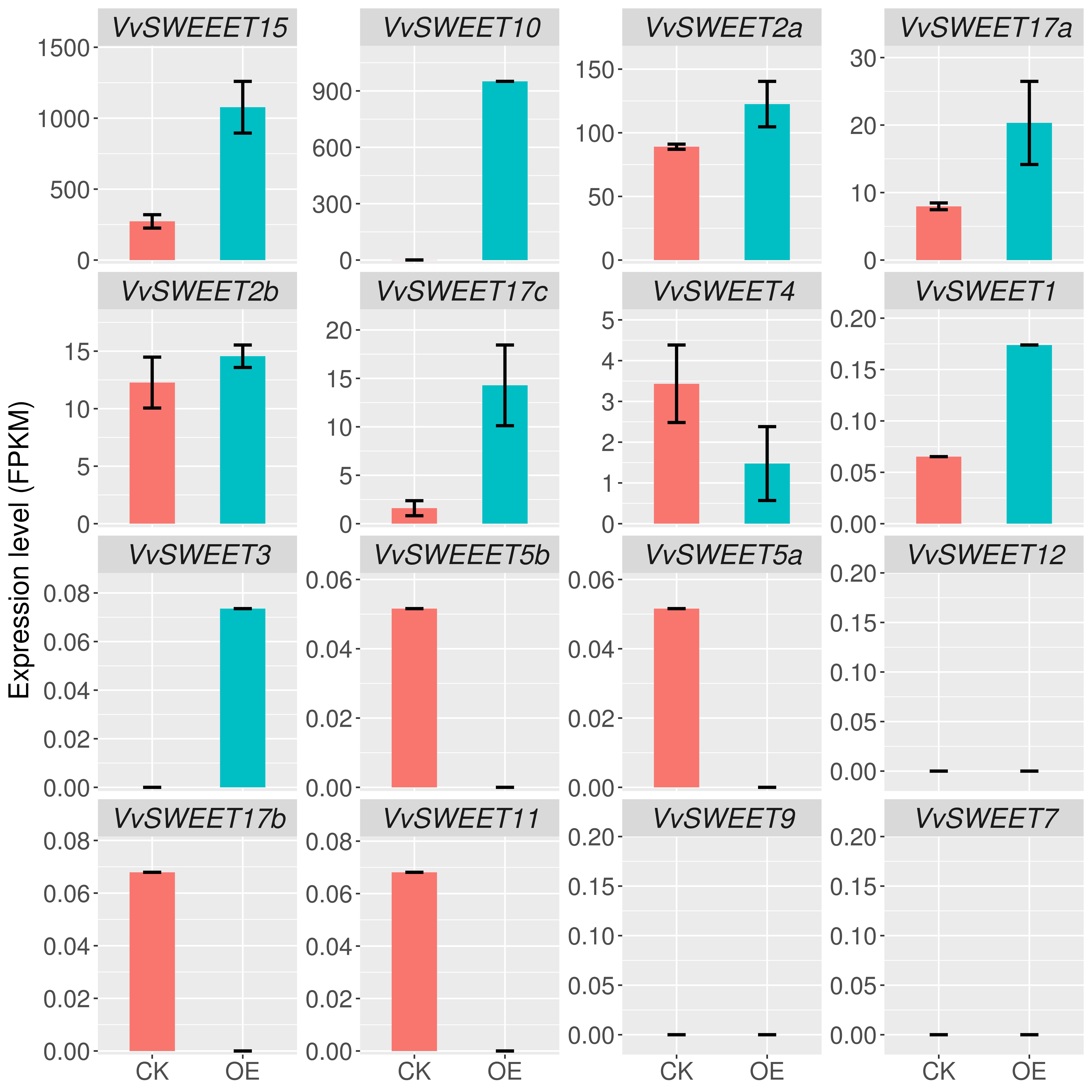

Supplement: Supplementary file 1 [file genes-10-00255-s001.zip › Fig. S7.jpg]

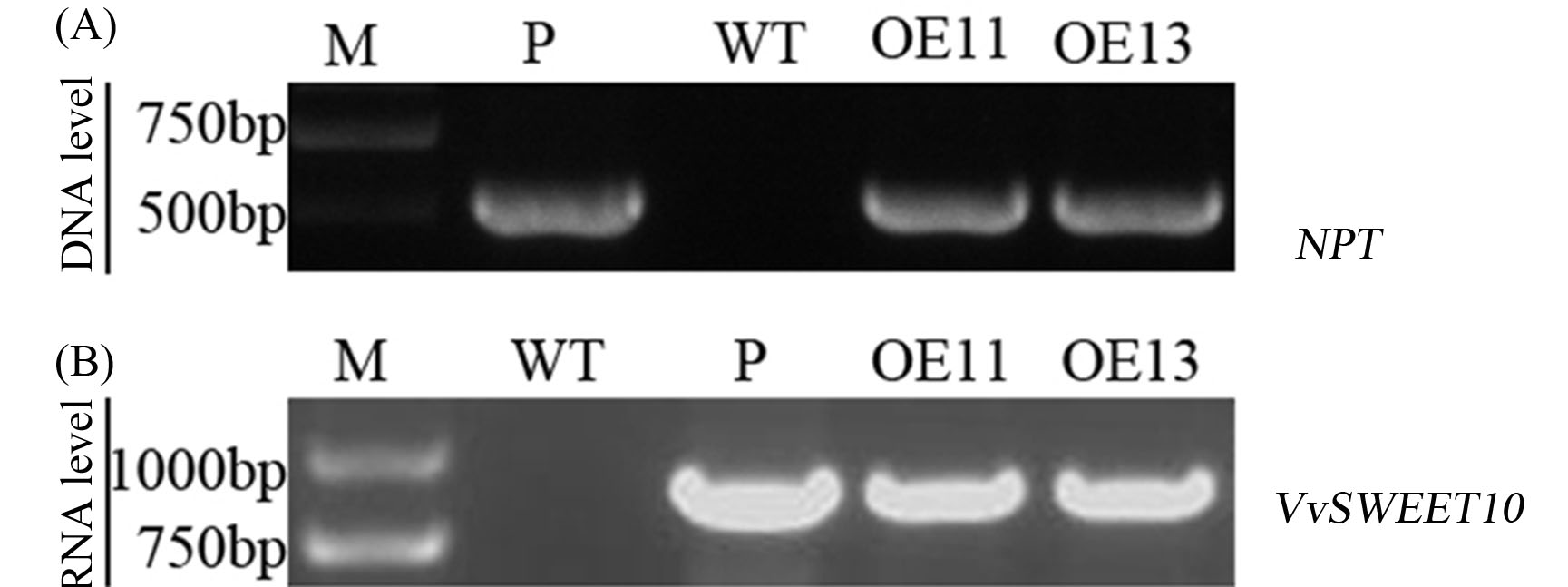

Supplement: Supplementary file 1 [file genes-10-00255-s001.zip › Fig. S8.tif]
